# Accentuation of suicides but not homicides with rising latitudes of Greenland in the sunny months

**DOI:** 10.1186/1471-244X-9-20

**Published:** 2009-05-08

**Authors:** Karin S Björkstén, Daniel F Kripke, Peter Bjerregaard

**Affiliations:** 1 Karolinska Institutet, SLSO, Psykiatri Södra Stockholm, Sköntorpsvägen 29, 2 tr., SE-120 38 Årsta, Sweden; 2 Scripps Clinic Sleep Center, 10666 North Torrey Pines Road, La Jolla, CA 92037, USA; 3 National Institute of Public Health, University of Southern Denmark, Øster Farimagsgade 5A, 2nd floor, DK-1399 Copenhagen K, Denmark

## Abstract

**Background:**

Seasonal variation in suicides has been shown in many countries. We assessed the seasonality and the variation with latitude in suicides and homicides, and the impact of alcohol on the seasonality in suicides.

**Methods:**

Official computerized registers on causes of death in all Greenland during 1968–2002 were used. Sales data on beer from one of the major food store chains for July 2005–June 2006 were examined. Seasonal variation was assessed by Rayleigh's test for circular distributions.

**Results:**

There were a total of 1351 suicides and 308 homicides. The suicides rate varied from 4.2/100 000 person-years in 1971 to 128.4/100 000 person-years in 1987. The homicide rate varied from 2.1/100000 person-years in 1969–1970 to 34.8/100 000 person-years in 1988. Out of the 1351 suicides, 80.5% were committed by men and 19.5% by women. Median age was 25 years (n = 1351; Range 11–84 years). Violent methods of suicide were used in 95% of all cases (n = 1286). Out of the 308 homicide victims, 61% were men and 39% were women, and 13% were killed in multiple homicide events.

There was a significant seasonal variation with peaks in June and troughs in the winter in all suicide cases (n = 1351, r = 0.07; Z = 7.58, p < 0.001), in violent suicides (n = 1286; r = 0.07; Z = 6.97; p < 0,001), in suicides in men (n = 1087; r = 0.07; Z = 5.39; p < 0.002) , and in women (n = 264; r = 0.10; Z = 2.36; p < 0.05), but not in homicides nor in consumption of beer. There was a bi-phasic seasonal variation in suicide victims where an alcohol-related condition was included in the death certificate

Suicides were more concentrated in the summer months north of the Arctic Circle (n = 577, r = 0.09, Z = 4.45, p < 0.01) than south of it (n = 769, r = 0.07, Z = 3.76, p < 0.002) and most concentrated in North Greenland (n = 33; r = 0.35; Z = 4.11; p < 0.01), where 48% of suicides occurred during the period of constant light. When including astronomical twilight in the constant light period 82% occurred during this time.

**Conclusion:**

There was a concentration of suicides but not homicides in the summer months in all Greenland. The concentration was most pronounced at high latitudes.

## Background

Greenland, located at the latitudes 60–80°N is the most extreme of natural human habitats regarding seasonal variation of light. The location along with a fairly homogenous Inuit population and reliable statistics makes the country an excellent place for the study of the influence of natural light on human behavior, in this study suicide, homicide and alcohol intake. Greenland is politically a part of Denmark, but has had home rule since 1979.

The suicide rate in Greenland increased during the 1970's from a historically very low level to one of the highest levels in the world, 107 per 100000 person-years in 1990–1994 [1]. The increase has been most pronounced among teenagers and young adults. A rapidly increasing suicide rate has been reported from other areas going through radical changes like in Eastern Europe after the fall of communism and among aboriginal people confronted with modern life-style.

We have previously demonstrated that the vast majority of suicides in West Greenland are violent and peak in the summer when the Northern half of Greenland has constant day-light and the Southern half has extremely long days [2]. Depression has, however, been reported uncommon [3] and the majority of suicides seem impulsive rather than depressive.

The overall homicide rate in Greenland has been reported much higher than that of the other Nordic countries [4]. Homicides are almost exclusively impulsive and committed under the influence of alcohol according the annual police reports in Greenland. At several occasions, more than one person has been killed at the same time. The penal system is mild and tolerant. Local prisons allow considerable freedom with a possibility to keep a job and family relations in the community, even for those who are convicted with murder. Only very dangerous convicts are sent to security prisons or forensic psychiatric units in Denmark. The seasonality in homicides in Greenland has not been previously studied.

### Seasonality in suicide

Seasonality in suicides was described in the 19^th ^century [5,6]. In spite of most studies showing spring and summer peaks, it is a wide-spread belief that the peak occurs in late autumn and early winter in relation to darkness [7]. Spring and summer peaks have been demonstrated in the northern hemisphere in countries like West Greenland [2], Finland [8], Norway [9], Belgium [10], France [11], Italy [12], Japan [13], USA [14], Lithuania [15], Switzerland [16]. The southern hemisphere shows a mirror image: Spring and summer peaks in December Chile [17], in September–October in South Africa [18], in November in Queensland [19] and in October in Victoria [20]. Studies from the equator are scarce, but an absence of seasonal variation has been reported from Singapore [21] and also from places more distant from the equator like Los Angeles and Sacramento, California [22].

Gender differences, as well as differences between age groups and methods of suicide have been reported in a number of studies. Violent suicides, i.e. mainly hanging, shooting and jumping account for a large part of the seasonal variation in suicides [2,8,10,15,20]. A correlation between sunlight and suicide has been proposed by several researchers [23].

In our first study of seasonality in suicides in West Greenland, there were not enough cases to study the impact of latitude [2]. By expanding the study to cover about twice as many cases and all Greenland, the present study allows us to split data and analyze differences between the north and south of Greenland.

### Seasonality in homicide

The seasonal variation in homicides has been less investigated than that in suicides. In 1957–1995 in Finland, a significant correlation between homicides and violent suicides peaking in summer peak was found [24]. Violent crimes in 1991–1997 in Norway were shown to peak in May-June and October-November [25]. The seasonal pattern of violent crimes resembled that for some forms of suicide and for hospitalization for affective disorders, and varied with latitude. In Belgium [10], Hong Kong [26] and in Japan [13] an absence of seasonality in homicide has been reported. By using FBI crime reports from 1976–1979, summer peaks for rapes but an absence of seasonal peaks for homicide in the USA were shown [27]. Sexual abuse of children in Minneapolis has been reported to peak in the summer [28]. Spring and fall peaks for child murder [29] but not for homicides in general were reported from Erie County, New York, between 1973 and 1983 [30].

### Serotonin, impulsivity and violence

Impulsivity and violent methods are shared characteristics between violent suicides and homicides in Greenland. An altered serotonin turn-over may be the common denominator. Serotonin turnover in men and women varies with season [31]. Pronounced summer peaks in serotonin and its metabolites in jugular vein blood have been reported from south-eastern Australia [32]. The same study also showed that the rate of production of serotonin in the brain was reported directly related to the prevailing duration of bright sunlight and rose rapidly with increased luminosity. Low levels of the serotonin metabolite 5-Hydroxyindoleacetic acid (5-HIAA) have been found in cerebro-spinal fluid (CSF) from patients who attempted suicide [33]. Similar inverse correlation between impulsive, externally directed aggressive behavior and CSF-5-HIAA has been reported in a subgroup of violent offenders [34]. Whole blood serotonin has been reported to correlate to violence among men but not women [35]. A decreased serotonin transporter function mediated by the s allele of 5-HTTLPR polymorphism has been associated with suicidal behaviour and violent suicides [36].

### Alcohol

Drinking habits vary widely with cultural, religious and socio-economic factors. Alcohol intake interferes with serotonin turn-over and is often involved in suicide, homicide [37] and other violent behaviour. A central serotonergic deficit has been reported in patients with early onset male alcoholism, [38]. The seasonality of alcoholism and alcohol-related suicides has not been carefully studied. An over-representation of suicide deaths in alcoholics during the second quarter of the year has been reported from Sweden [39].

Our previous study in West Greenland that showed increased alcohol consumption in the 70'ies and 80'ies somewhat parallel to the increase in suicides raised the question whether the summer suicides would correspond to increased drinking in the summer, when the suicide peak occurred.

The aim of this current work was to assess

Whether there is a seasonal variation in all Greenland regarding

• suicide in general and in persons with alcoholism

• homicide

• alcohol consumption

• whether there is an influence of latitude in the seasonal variation in suicides and homicides.

## Methods

This study covers all inhabitants in all Greenland: West, North and East Greenland during the 35 year period 1968–2002.

### Suicides and homicides

Official computerized registers on causes of death in Greenland were used. From 1967, all deaths in Greenland were collected systematically and registered according to the WHO International Classification of Diseases 8^th ^edition (ICD-8) and from 1994 the 10^th ^edition (ICD-10). In all cases of un-natural death, the police and the local doctor discuss the case in order to determine the cause of death. The local doctor has access to all patient records. Autopsies are rarely performed. Data were collected regarding date of birth, age, gender, date and place of suicide or homicide, and country of birth (Greenland or Denmark).

Only definite suicides and homicides, not possible cases, were included. Method of suicide was recorded according to ICD-8 for the years 1968–1993 and ICD-10 for the years 1994–2002. Intentional self-poisoning by gas, drugs or other ingested substances were classified as non-violent suicides. Hanging, shooting, intentional drowning, cutting and piercing with sharp objects, and jumping from high places were classified as violent suicides. Homicide data refer to victims, not to perpetrators.

All cases where the second or third position of the death certificate included psychiatric diagnosis were also identified.

### Population

For the earlier years studied, population statistics were obtained from the Danish statistical year books from 1974, 1975 and 1977 [40-42] and for the later years studied, computerized population registers from the National Institute of Public Health in Copenhagen and Statistics Greenland's website  were used.

In 1968, the first year of the study, 84% of the 45,639 inhabitants were born in Greenland and 16% were born in Denmark. There were slightly more men than women in the population born in Greenland throughout the years of this study. In the Danish-born population, there were about twice as many men than women. The excess of Danish men is mainly caused by immigration of skilled work force.

The population had gradually increased to 56,542 inhabitants in 2002, the last year of the study, 88% of whom were born in Greenland and 12% outside Greenland, almost exclusively in Denmark.

Although there is a substantial European genetic admixture, birthplace Greenland serves as a proxy for Inuit ethnicity and birthplace Denmark as a proxy for Danish Ethnicity, although there are exceptions on both sides.

By the end of this study, in 2002, the majority (about 92%) lived in West Greenland, an area covering latitudes 60–73°N, including the capital Nuuk. About 1.5% of the population lived in the isolated town of Qaanaaq (Thule) at 77°N in North Greenland covering latitudes 70–80°N, and about 6% of the population lived in East Greenland, mainly in Tasiilaq (Ammassalik) at 66°N and Illoqqortoormiut at 70°N.

The Arctic Circle at Latitude 66.7°N splits the country approximately in half. In 1990, 55% or 30807 persons lived south of the Arctic Circle and 45% or 23932 persons lived north of the Arctic Circle.

### Seasonal variation in light in Greenland

Daylight varies widely around the year and with latitude. The day-length changes gradually over the 20° from Cape Farewell in the south to Kap Morris Jesup in the north.

In Qaanaaq, at latitude 77°N in the heart of North Greenland, the sun rises on April 20^th ^and stays continually above the horizon until it sets on August 22^nd^. The sun sets for the winter on October 28^th ^to rise on February 13^th^. When astronomical twilight is included, the bright period starts on March 7^th ^and ends on October 8^th^

South of the Arctic Circle, there is no mid-night sun, but very long days and twilight periods occur in the summer. The situation in the winter is the reverse with very short days.

When snow and ice are present, they may contribute to very strong reflection of light, actually in extreme cases to the extent of causing acute blindness, if eye covers or sun-glasses are not used.

### Alcohol

Beer represents more than 70% of alcohol intake in Greenland. Official statistics on alcohol sale taxes were used to obtain alcohol consumption expressed as litres of pure alcohol per person above 14 years of age per year. Data from 1968–1974 were obtained from the Ministry of Greenland's official year-books and data for the years 1975–2002 from . Official statistics cannot be used for estimation of seasonal consumption since, due to weather conditions, supplies are transported when possible, i.e. mainly during the summer.

Detailed sales data are secret for business reasons. One of the major food store chains has generously and under discretion provided sales data on beer for the period of July 2005–June 2006 for all Greenland. The volume sold was given in arbitrary units. Those data have been used as a proxy for seasonality in alcohol consumption.

### Ethics

Ethical approval was obtained from the Commission for Scientific Research in Greenland.

### Statistical methods

Suicide and homicide rates per 100 000 person-years (all ages) were calculated for each year of the study.

Seasonal variation was assessed by Rayleigh's test for circular distributions [43]. The results of the calculations of Rayleigh's test are expressed by Rayleigh's "r" (which varies inversely with the circular dispersion of the data) and by Rayleigh's "z" (= n × r^2^) that can be used for testing the null hypothesis of no population mean direction, and the p value. The exact dates were used for calculations of seasonality. For calculation of seasonality of beer sales, arbitrary units per month were used. Data from all or selected years were pooled due to the small number of cases.

To assess the influence of latitude, suicide and homicide data were split at the Arctic Circle (Latitude 66.7°) and analyzed separately for cases on either side of the Arctic Circle. In addition, suicide data were also analyzed separately from North Greenland, the northernmost natural human habitat in the world at latitudes 70–80°. It was not possible to obtain alcohol sales data split at the Arctic Circle.

The Spearman Rank Correlation was used to assess the correlation between yearly alcohol consumption in the population and the yearly incidence of suicides and homicides respectively.

Calculations were made in the computer programme Statistica version 7 and Excel version 2003.

## Results

**Figure 1:**
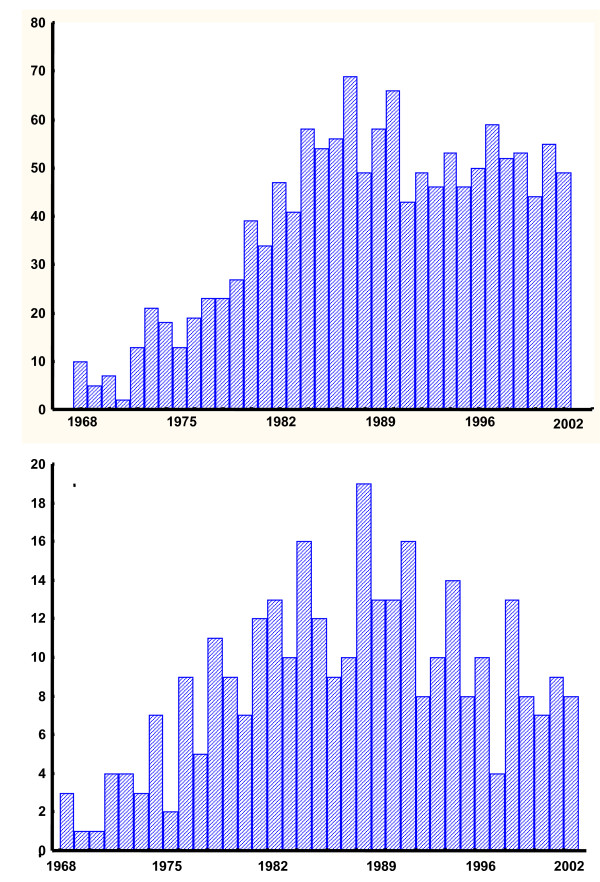
**Suicides and homicides plotted against year**. Fig 1a and b show the number of suicides (1a- upper) and the number of homicides (1b lower) in 1968–2002 plotted against year. Please note that the scales on the Y-axes are different.

There were a total of 1351 suicides and 308 homicides during the 35 year period 1968–2002. Fig 1a shows the total number of suicides per year and Fig 1b shows the total number of homicides per year.

**Figure 2:**
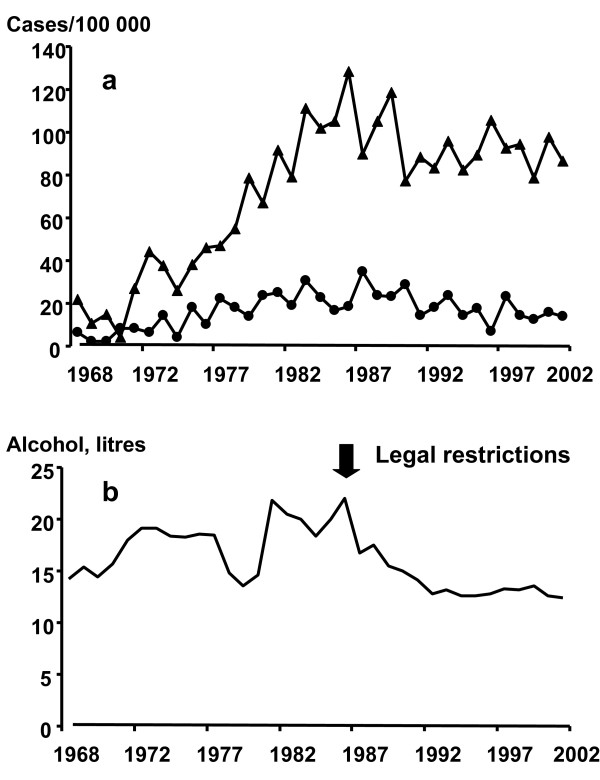
**Suicide rate, homicide rate and alcohol consumption plotted against year**. Fig 2a shows the suicide and the homicide rates in 1968–2002 expressed as cases per 100000 persons per year plotted against year. Fig 2b shows the alcohol consumption for the years 1968–2002 expressed in litres per person >14 years of age per year.

Fig 2a shows the suicide and homicide rates expressed as suicides per 100000 person- years. From a minimum of 2 cases (4.2 suicides/100 000 person-years) in 1971, there was an increase to a maximum of 69 cases (128.4 suicides/100 000 person-years) in 1987 and 66 cases (118.8 suicides/100 000 person-years) in 1990, and 43–59 cases or (77.3 – 105.4 suicides/100 000 person-years) per year thereafter.

For homicides, there was an increase from a minimum of 1 homicide (2.1/100000 person-years) in 1969–1970 to a maximum of 19 homicides (34.8/100 000 person-years) in 1988 and 16 homicides (28.8/100 000 person-years) in 1991, and 4–13 (7.1–23.5/100 000 person-years) homicides per year thereafter.

Fig 2b shows the yearly alcohol consumption expressed as litres per person >14 years of age.

### Demographic data of suicide and homicide cases

There were 1087 suicides in men (80.5%), the vast majority (n = 1020) being born in Greenland, and the remaining being born in Denmark (n = 66). Country of birth was missing for one man. There were 264 suicides in women (19.5%). 257 women being born in Greenland and 7 women being born in Denmark.

Persons in upper teens and young adults were heavily over-represented among the suicide cases. Median age was 25 years (n = 1351; Range 11–84 years). In men, median age was 25 years (n = 1087; Range: 11–84 years) and in women 26 years (n = 264; Range 12–80 years).

In 391 out of the 1351 cases (29%), the death certificate included a psychiatric diagnosis. In 214 cases (15.8%), there was a diagnosis of alcoholism or alcohol intoxication; two cases also had a diagnosis of psychosis. In only 52 cases (3.8%), there was a diagnosis of affective disorder, either unspecified or in the depressive state. In 104 cases, there was a diagnosis of psychosis. In addition to the 104 cases (7.7%), there were two with alcoholism and psychosis.

Out of the 308 homicide victims, 187 (61%) were men and 121 (39%) were women. There were no women but 15 men born in Denmark who were killed. There were thus 172 men and 121 women born in Greenland that died by homicide.

Homicide victims were generally older than suicide victims. Median age was 34.5 years (n = 308; Range 0–81 years). In men, median age was 35 years (n = 187; Range 0–77 years) and in women 34 years (n = 121; Range 0–81 years).

### Method of suicide and homicide

Violent methods of suicide were used in 95% of all cases (n = 1286); Hanging 46% (n = 625), shooting 37% (n = 573), jumping from heights 2% (n = 23), cutting with sharp objects <1% (n = 6) and drowning 4% (n = 50) and unspecified (<1%) n = 9. Less than 5% committed suicide by poisoning. Men used violent methods in 97% (n = 1059) and women in 86% (n = 226) of the cases.

In 119 of the 308 homicide cases, victims were killed by shot-guns or explosives, 95 by cutting or piercing objects, 23 by hanging, strangulation or suffocation, 21 by fights without weapons, 5 by drowning, 4 were killed with blunt objects, and the remaining 41 cases were classified as killed by other or non-specified method. In 17 homicide events, several persons were killed in the same event (13 events; 2 victims; 3 events; 3 victims, 2 events; 4 victims). Altogether, 39 persons or 13% were killed in multiple homicide events. In 269 cases or 87%, a single person was killed in the same event.

### Season of suicide and homicide

**Figure 3:**
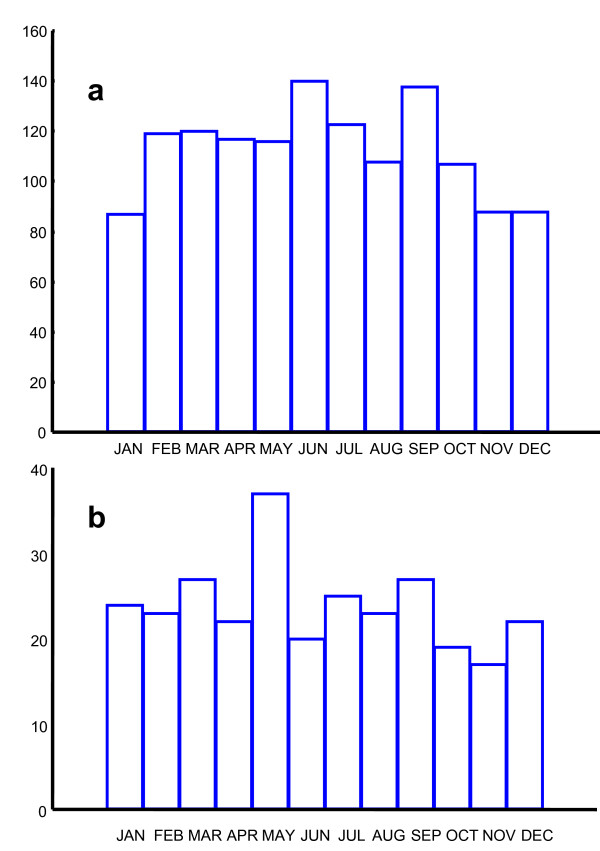
**Monthly distributions of suicides and homicides**. The monthly distribution of all suicides (n = 1351) is shown in Fig 3a and all homicides events (n = 286) in Fig 3b. Please note that the scales on the Y-axes are different.

The monthly distribution of all suicide deaths is shown in Fig 3a. There was a significant seasonal variation in all cases (n = 1351, r = 0.07; Z = 7.58, p < 0.001), the calculated annual peak occurring on June11th and the trough in November-January. There was a significant seasonal variation in violent suicides (n = 1286; r = 0.07; Z = 6.97; p < 0,001). Because of the smaller number of subjects, but not because of the smaller r value representing seasonality, there was no significant seasonality in non-violent suicides (n = 65; r = 0.14; Z = 1.41; p = n. s.). The seasonal variation of suicides was statistically significant in men (n = 1087; r = 0.07; Z = 5.39; p < 0.002) and in women (n = 264; r = 0.10; Z = 2.36; p < 0.05.) with calculated peaks in June.

The monthly distribution of all homicide events (n = 286) is demonstrated in Fig 3b. In calculations of seasonality in homicides, multiple homicide events were regarded as one case. The calculated annual peak occurred on May 2^nd^, but the seasonal variation in homicides was not significant (n = 286; r = 0.08; Z = 1.67; p < 0.10). When data were split at the Arctic Circle, the seasonal variation was not significant on either side (South n = 162; r = 0.10; Z = 1.75; p < 0.20; North n = 145; r = 0.07; Z = 0.76; p < 0.20).

### Season of suicide in relation to latitude

**Figure 4:**
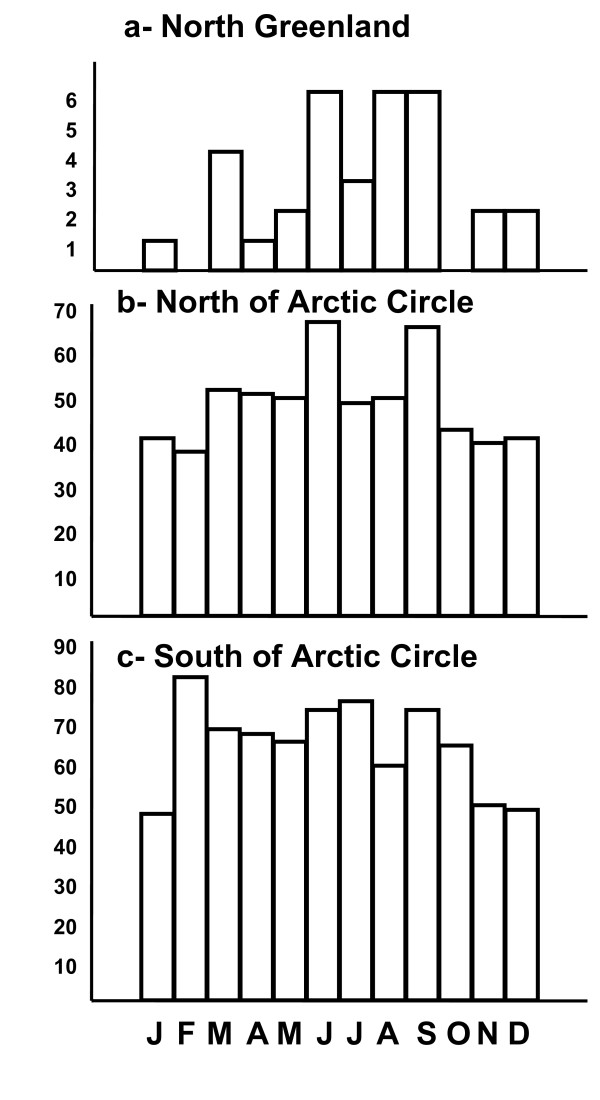
**Monthly distributions of suicides at different latitudes**. Fig 4a (upper) shows the monthly distribution of suicides in North Greenland (n = 33); Fig 4b (middle) north of the Artic Circle (n = 577) and Fig 4c (lower) south of the Arctic Circle (n = 769).

In spite of the small number of suicides (n = 33) in the province of North Greenland, we found a strong concentration of suicide deaths during the bright season, the annual peak occurring June 10th. Rayleigh's test (n = 33; r = 0.35; Z = 4.11; p < 0.01) as demonstrated in Fig 4a. Sixteen (48%) out of the 33 suicides occurred during the period of constant sun on April 20^th ^to August 22^nd ^(33% of the year). When including astronomical twilight in the period of constant sun, i.e. from March 7^th ^to October 8^th^, 27 (82%) out of 33 suicides occurred in constant light.

In the 33 suicide cases in North Greenland, 15 cases (45%) had a psychiatric diagnosis. None had a diagnosis of affective disorder. In ten of the cases (30%), alcohol contributed to the suicide, and one of those also suffered from schizophrenia. In four additional cases (12%) that occurred on March 26^th^, May 1^st^, June 28^th^, and August 15^th^, psychosis not otherwise specified was noticed in the death certificate.

When data were split at the Arctic Circle, a significant seasonality in suicide deaths was found on both sides of the Arctic Circle. The concentration of suicides to the bright months of the year was more pronounced north of the Arctic circle, as shown in Fig 4b, the calculated annual peak occurring on June 30^th ^(n = 577, r = 0.09, Z = 4.45, p < 0.01).

Fig 4c shows the monthly distribution of suicides South of the Arctic circle, the calculated annual peak occurred somewhat earlier on May 27^th ^and somewhat less concentrated as shown by the smaller 'r' value (n = 769, r = 0.07, Z = 3.76, p < 0.002). Data about location of suicides were missing in 5 cases.

### Alcohol

The yearly alcohol consumption expressed as litres per person >14 years of age is shown in Fig 2b below the suicide and homicide figures. Yearly alcohol consumption was about 14–15 litres per person above age 14 years during 1968–1972. Thereafter, there was an increase to about 18–19 litres per year in 1973–1978. After a three year decrease to about 13–14 litres per year, consumption increased to 18–22 litres per year during 1982–1987. The high consumption led to legal restrictions in alcohol sale, that resulted in a decrease to about 14–17 litres per year during 1988–1992. Since 1993, the alcohol consumption has stabilized itself at a level about 12–13 litres per year.

There was no statistically significant correlation between the yearly alcohol consumption and either suicides or homicides.

In 214 of the 1351 suicide cases (15.8%), the death certificate showed that alcohol had contributed to death. Of these 214 cases, 204 persons were born in Greenland and 10 were born in Denmark. Median age for all was 28.5 years (Range 14–66 years; n = 214). In men, median age was 28 years (Range 14–65 years, n = 172) and in women 29 years (Range 15–66 years; n = 42).

**Figure 5:**
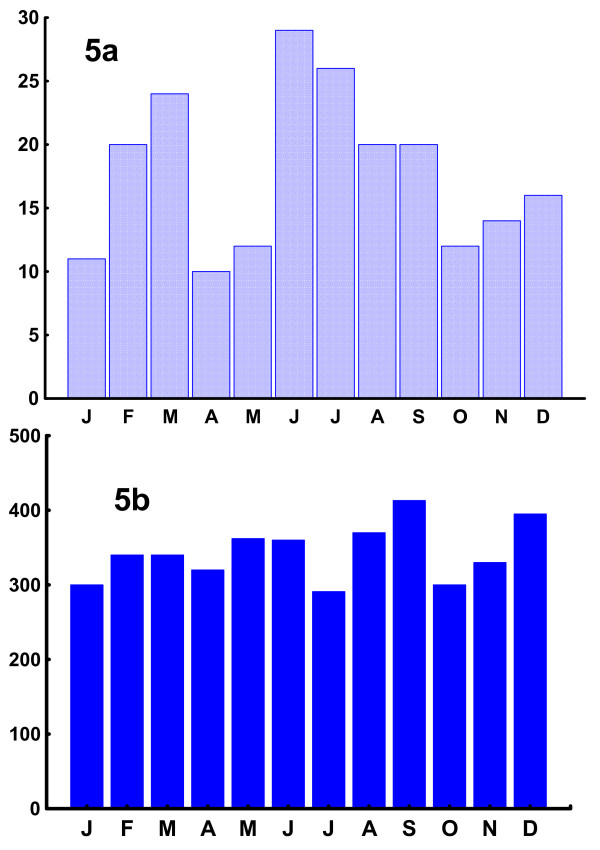
**Monthly distributions of alcohol-related suicides and of alcohol sale**. Fig 5a shows the monthly distribution of suicides where alcohol played a role (n = 214). Fig 5b shows the monthly sale of beer sale (arbitrary units) for the period of July 2005–June 2006.

The seasonal distribution of alcohol-related suicides showed a bi-phasic curve with fewer suicides from October – January and in April and May, as shown in Fig 5a. There was no statistically significant seasonal variation in beer sale for the period of July 2005–June 2006 (n = 4121, r = 0.01; Z = 0.93; p = n.s.) Fig 5b.

## Discussion

One of the new findings in this study is that the suicides were more concentrated in the summer in the north compared to south of the Arctic Circle and extremely concentrated in North Greenland. Out of the 33 suicides in North Greenland (latitude 77°N) 48% occurred in the period of constant sun or 82% if the constant sun period included astronomical twilight. This supports the idea of a correlation between sun-shine and suicide. None of the 33 suicide cases in North Greenland had a diagnosis of affective disorder but four persons had a diagnosis of psychosis not otherwise specified. All those four persons committed suicide during the period of constant sun. It is possible that they suffered from a mixed affective state or delirium triggered by sleeplessness in the bright summer night. People living at high latitudes need extreme flexibility in light adaptation. During the long periods of constant light, it is crucial to keep some circadian rhythm to get enough sleep and sustain mental health. A weak serotonin system may cause difficulties adapting to life at high latitudes.

This study showed that homicide deaths increased non-significantly in the spring, and that that the rate was high compared to other Nordic countries. The small sample may be the reason for lack of seasonality.

This study covering all of Greenland and seven additional years confirmed our finding from West Greenland [2] that 1) the general suicide rate was very high, and 2) that the vast majority of suicides were violent, and that summer peaks were present in 3) all suicides, 4) in violent suicides and 5) in suicides in men. In the present study with a larger sample, we found a significant summer peak also in women. Since 86% of women in Greenland in contrast to those in many other countries used violent methods, this finding is in agreement with other studies showing that violent methods account for an important part of the seasonality in suicides.

We found no seasonal variation in the sale of beer. One shortcoming of the sales data are that they were collected later than the years of suicides, and that they cover only one year and beer only from one of the major food store chains. An advantage is that there are very few shops, and that the data shared with us can be expected to be reliable. Getting such data from a country with a large variety of shops and a population travelling and frequently shopping abroad would be less reliable.

The bi-phasic seasonal variation of suicides where alcohol has been involved (n = 214) is difficult to interpret, and more studies and larger materials are required to elucidate whether seasonality in alcohol-related suicides is an issue. There is a considerable variation in how additional diagnoses are recorded in death certificates, and the diagnosis were not established for research purposes. There are few studies of seasonality in suicides in alcoholic patients, but a Swedish study of suicides in 99 alcoholic men diagnosed at a university department showed a suicide peak during the second quarter of the year.

This material of 1351 definite suicides included only 391 cases (29%) with a known diagnosis of a psychiatric diagnosis in the death certificate. Since depression is considered a major reason for suicide, the 52 cases (3.8%) with an affective diagnosis is an extremely low figure compared to other studies. The prevalence of depression, diagnosed with western criteria may actually be low, as shown by Lynge [3]. Other reasons for the low figure may be that Western diagnostic criteria and tradition of expressing feelings does not fit the Inuit culture very well.

## Conclusion

The suicide rate in Greenland is one of the highest in the world. Suicides were almost exclusively violent with significant summer peaks when there is either mid-night sun or very long days. The suicides were more concentrated around the summer months at higher latitudes. At about 77°N, 82% of the suicides occur during the period of constant day.

In 29% of the suicide cases, there was a psychiatric diagnosis in the death certificate, however rarely depression (3.8%).

Homicide deaths showed a non-significant increase in spring, and the rate was high compared to other Nordic countries.

There was a bi-phasic seasonal variation for suicides related to alcohol, but no seasonal variation in consumption of beer.

Light is only one of many factors in the complex tragedy of suicide, but this study shows that there is a possible relationship between light and suicide.

## Competing interests

The authors declare that they have no competing interests.

## Authors' contributions

KSB conceived of the study, carried out all calculations and drafted the manuscript. PB provided the data and special expertise on Greenland. DFK provided expertise on light and biological rhythms. All authors read and approved the final manuscript.

## Pre-publication history

The pre-publication history for this paper can be accessed here:


